# Inhaled ambient-level traffic-derived particulates decrease cardiac vagal influence and baroreflexes and increase arrhythmia in a rat model of metabolic syndrome

**DOI:** 10.1186/s12989-017-0196-2

**Published:** 2017-05-25

**Authors:** Alex P. Carll, Samir M. Crespo, Mauricio S. Filho, Douglas H. Zati, Brent A. Coull, Edgar A. Diaz, Rodrigo D. Raimundo, Thomas N. G. Jaeger, Ana Laura Ricci-Vitor, Vasileios Papapostolou, Joy E. Lawrence, David M. Garner, Brigham S. Perry, Jack R. Harkema, John J. Godleski

**Affiliations:** 1 Department of Environmental Health, Harvard T.H. Chan School of Public Health, Boston, MA USA; 2 Department of Physiology, Diabetes and Obesity Center, School of Medicine, University of Louisville, 580 South Preston Street, Delia Baxter Building, Room 404B, Louisville, KY 40202 USA; 3 School of Medicine, University of São Paulo, São Paulo, Brazil; 4 Faculty of Public Health, University of São Paulo, São Paulo, Brazil; 5 Federal University of São Paulo, São Paulo, Brazil; 6 Faculty of Health and Life Sciences, Oxford Brookes University, Oxford, UK; 7 Department of Pathobiology, Michigan State University, East Lansing, MI USA

**Keywords:** Particulate matter, Secondary organic aerosol, Baroreflex, Heart rate variability, Autonomic, Arrhythmia, Traffic, Respiratory, Cardiopulmonary

## Abstract

**Background:**

Epidemiological studies have linked exposures to ambient fine particulate matter (PM_2.5_) and traffic with autonomic nervous system imbalance (ANS) and cardiac pathophysiology, especially in individuals with preexisting disease. It is unclear whether metabolic syndrome (MetS) increases susceptibility to the effects of PM_2.5_. We hypothesized that exposure to traffic-derived primary and secondary organic aerosols (P + SOA) at ambient levels would cause autonomic and cardiovascular dysfunction in rats exhibiting features of MetS. Male Sprague Dawley (SD) rats were fed a high-fructose diet (HFrD) to induce MetS, and exposed to P + SOA (20.4 ± 0.9 μg/m^3^) for 12 days with time-matched comparison to filtered-air (FA) exposed MetS rats; normal diet (ND) SD rats were separately exposed to FA or P + SOA (56.3 ± 1.2 μg/m^3^).

**Results:**

In MetS rats, P + SOA exposure decreased HRV, QTc, PR, and expiratory time overall (mean effect across the entirety of exposure), increased breathing rate overall, decreased baroreflex sensitivity (BRS) on three exposure days, and increased spontaneous atrioventricular (AV) block Mobitz Type II arrhythmia on exposure day 4 relative to FA-exposed animals receiving the same diet. Among ND rats, P + SOA decreased HRV only on day 1 and did not significantly alter BRS despite overall hypertensive responses relative to FA. Correlations between HRV, ECG, BRS, and breathing parameters suggested a role for autonomic imbalance in the pathophysiologic effects of P + SOA among MetS rats. Autonomic cardiovascular responses to P + SOA at ambient PM_2.5_ levels were pronounced among MetS rats and indicated blunted vagal influence over cardiovascular physiology.

**Conclusions:**

Results support epidemiologic findings that MetS increases susceptibility to the adverse cardiac effects of ambient-level PM_2.5_, potentially through ANS imbalance.

**Electronic supplementary material:**

The online version of this article (doi:10.1186/s12989-017-0196-2) contains supplementary material, which is available to authorized users.

## Background

Ambient PM_2.5_ exposure is tied to cardiovascular disease morbidity and mortality. Furthermore, preexisting cardiovascular disease and diabetes strengthen the link between PM_2.5_ exposure and acute cardiac events such as arrhythmia, cardiac arrest, and heart failure decompensation [1]. Studies involving direct exposure of animal disease models have added biological plausibility while implicating ANS imbalance, thrombosis, inflammation, and oxidative stress as key mediators. The dose-effect relationship for PM_2.5_-induced cardiac pathophysiology, its modifying factors, and the underlying mechanisms at real-world exposure levels remain unresolved.

MetS afflicts one third of U.S. adults and is a cluster of at least three of the following risk factors—systolic/diastolic blood pressure (BP) ≥130/85 mmHg, elevated triglycerides, high fasting glucose, abdominal obesity, and low circulating high density lipoprotein [2]. In MetS subjects researchers have found exaggerated cardiovascular inflammation [3] as well as increased BP and altered heart rate variability (HRV) in association with moderate increases in PM_2.5_, suggesting pathogenic sympathetic nervous system dominance, a state of enhanced sympathetic influence relative to opposing parasympathetic influences over the cardiovascular system [4, 5].

In contrast, PM_2.5_ exposure at levels beyond U.S. extremes (356 μg/m^3^) in a rat model of MetS decreased BP and heart rate while increasing HRV, suggesting relative vagal dominance and significantly diverging from the sympatho-excitatory effects of PM_2.5_ in normal diet-fed rats at similar exposure levels [6]. Yet, PM_2.5_-exposed MetS rats had enhanced epicardial adipose tissue inflammation and oxidative stress compared to normal diet PM_2.5_-exposed rats [7]. Given recent findings that preexisting cardiometabolic risk factors may not enhance the association between PM_2.5_ and cardiovascular mortality in humans [8], it remains unclear if and how MetS might actually alter susceptibility to PM_2.5_ exposure at near-ambient levels. To further explore whether MetS increases susceptibility to PM_2.5_, we investigated the pathophysiologic effects of repeated exposure to traffic-derived PM_2.5_ at ambient levels in a rat model of MetS characterized by hypertension, hypertriglyceridemia, hyperglycemia, and insulin resistance induced by 8-week dietary fructose supplementation [6, 9, 10]. We hypothesized repeated exposure to traffic-derived PM_2.5_ would cause cardiovascular dysfunction in MetS rats distinct from similarly-exposed ND rats. We analyzed arterial pressure and electrocardiograms (ECG) continuously for changes in HRV, cardiac arrhythmia, and spontaneous BRS over a three-week exposure to either FA or PM_2.5_ from an urban highway tunnel.

## Methods

### Animals

Adult male Sprague Dawley (SD) rats (250–300 g; Taconic Farms Inc., Rennselaer, NY) were housed and treated in accordance with National Institute of Health guidelines for the care and use of laboratory animals. All protocols were approved by the Harvard Medical Area Institutional Animal Care and Use Committee. Normal diet (ND; irradiated PicoLab Rodent Diet 20 5053, Lab Diet, St. Louis, MO) rats were catheterized in the descending abdominal aorta with BP telemeters (*n* = 8; DataSciences International, St. Paul, MN, PAC-40) whereas high fructose diet (HFrD) rats were implanted with aortic BP + ECG telemeters in a Lead II configuration (*n* = 12, DSI C50-PXT) at Taconic by trained surgical staff and shipped two weeks later to our mobile laboratory described previously [11]. We previously characterized effects of P + SOA exposure in these ND-fed rats on BP and heart rate (HR) [12] and respiratory patterns [13]. Here, we used the same exposure procedures to compare effects of P + SOA on a rat MetS model to these ND-fed rats with more advanced endpoints, including HRV, BRS, and BP indices of cardiac performance, with additional ECG analyses in MetS rats. SD rats fed an 8-week 60% fructose diet (HFrD) bear several clinical features of MetS and thus provide a useful animal model of this syndrome in humans. The MetS group (*n* = 12) received HFrD (60% fructose by mass; TD.89247, Harlan Laboratories, Madison, WI) for 8 weeks prior to and throughout inhalation exposure. At the conclusion of the exposure study, blood was collected from MetS rats, centrifuged for serum, aliquoted, stored at −80 °C, and subsequently analyzed for serum glucose by spectrophotometry at IDEXX Laboratories, Inc. (North Grafton, MA, USA) on an Olympus 5400 chemical analyzer.

### Particulate exposure

We applied two inhalation exposure controls—a 5-h HEPA-filtered air (FA) baseline in ND and MetS rats, and FA groups for each diet. After two 2-h FA exposure acclimations over two days, and a 5-h baseline, MetS (*n* = 12; 127 days old) and ND (*n* = 8; 100 days old) rats were assigned evenly and randomly (by weight) for exposure to FA or combined primary traffic PM_2.5_ (<2.5 μm aerodynamic diameter) and photochemically-derived secondary organic aerosols (P + SOA) from a major northeastern U.S. traffic tunnel plenum for 12 days, 5 h/day, 4 days/week over 3 weeks. Exposure tubes were maintained at 1.5 L/min continuous air flow and monitored with plethysmographic transducers (Buxco Electronics, Wilmington, NC) as previously described along with the exposure system [11, 13]. Aerosol was oxidized in a photochemical reaction chamber with enough ozone (O_3_) added to titrate vehicular nitric oxide (NO) exhaust and expedite stable secondary particulate generation [14]. After a 4-h residence, aerosol was delivered continuously via two parallel denuders [15]—reducing gaseous pollutant concentrations by 80–90%—to animal exposure tubes. ND and MetS rats received inhalation exposures in August 2011 and October 2012, respectively. Particle size distribution and concentrations were measured using a scanning mobility particle sizer (SMPS Model 3934, TSI Inc., Shoreview, MN) and a condensation particle counter (CPC Model 3785, TSI). We measured O_3_ by UV photometry (2B Technologies Inc., Boulder, CO), NO and nitrogen oxides (NOx) by chemiluminescence (Model 42C, Thermo Scientific, Waltham, MA), and carbon monoxide (CO) by infrared absorbance (Model 48, Thermo).

### ECG and BP analyses

For time-series data (except non-normal arrhythmia counts), each rat’s change from its own baseline (“delta”) was calculated on each exposure day. ECG and BP waveforms were continuously collected during exposure and analyzed in 5-min segments. ECG morphology and HRV were derived using ecgAuto, v3.3 (Emka Technologies, Paris, France) and a library of 199 representative beats with configurations previously described for low frequency (LF) and high frequency (HF) [16]. Time-domain measures included root mean square of successive differences (RMSSD), standard deviation of normal interbeat intervals (SDNN), and coefficient of variation (CV)—rate-normalized SDNN. Time domain parameters for ND rats were generated from systolic pressure peak intervals identified by ecgAuto—an approach we validated in air and PM_2.5_-exposed MetS rats (Additional file 1: Table S2). To control for atrial premature beats (APBs), we removed inter-beat intervals shortened by >18% from the average of 3 preceding and subsequent intervals similar to human studies [17, 18]. Baseline data were also analyzed by non-linear HRV methods (Additional file 1: Tables S3 and S4). We classified arrhythmias in MetS rats by treatment-blind ECG inspection, verification in BP, and confirmation by a second investigator using previously described criteria [19] for all but APBs (>18% RR shortening vs. neighboring 6 RRs, normal PR) and non-conducted APBs (ncAPB; dropped QRS and >18% PP shortening vs. prior 4 PPs). Non-conducted P-wave arrhythmias were differentiated as either ncAPB (premature P wave unfollowed by a QRS), second degree AV block Mobitz I events (a P at a normal interval from the prior P and accompanied by a ≥ 2-fold increase in RR interval but preceded by PR interval prolongation within the prior 4 normal QRS complexes or a PR interval shortening in the first conducted QRS complex thereafter), second degree AV block Mobitz II events (un-prolonged PR over the four prior QRS complexes, normal PR interval in the first conducted QRS complex thereafter), or advanced AV block (same criteria as Mobitz II, but RR ≥ 3-fold increase). ECGs on all exposure days were examined for all arrhythmias except APBs, which were identified on half of exposure days selected at random (Baseline and days 1, 4, 6, 8, 11, 12). BP was analyzed as described previously [20], including aortic *dP/dt*_*max*_, an indirect index of left ventricular contractility and predictor of death and decompensation in heart failure patients [21, 22]. After exclusion of ectopic beats, we calculated spontaneous BRS slope by the “sequence method” [23] in ecgAUTO, selecting inter-beat interval series (≥ 3) in which successive pairs of systolic peaks and subsequent beat intervals varied ≥0.50 mmHg and ≥0.30 ms, respectively, with a correlation ≥0.50. ECG morphology was analyzed as described [19], adding minimum ST slope (S min. Slope, during initial 1.5 ms after S) and ST amplitude (ST amp, mean at 2–4 ms after S). QT was rate-corrected by Fridericia formula or, only where specifically indicated, Bazzett’s formula.

Time series physiologic data are presented as change from baseline to allow consistent comparison to reported effects of much higher PM_2.5_ exposures in this same model of high fructose diet-induced MetS [6]. For time-series data where possible, daily differences in deltas between P + SOA and FA groups receiving the same diet are presented as single daily values to facilitate comparison to a prior companion study involving MetS rats and higher PM_2.5_ exposure concentrations [6].

### Ventilatory durations

We analyzed whole body plethysmographic data from MetS rats as previously detailed [13] to provide daily averages of inspiratory, expiratory, and total times (T_I_, T_E_, and T_T_), respiratory frequency (*f*), and respiratory pause from continuous 10-min averages.

### Statistics

Baseline data were compared between diets by student’s two-tailed t-test (Microsoft Excel), with *P* < 0.05 considered significant and all other analyses performed in SAS 9.3 (Cary, NC). We analyzed time-series deltas (each animal’s change during exposure from its own value at baseline) with linear mixed effects models (PROC MIXED) for daily or overall inhalant effects while controlling for day and selecting a random effects structure using AIC best fit criteria. Given their non-normality and longitudinality, we analyzed arrhythmia counts via generalized estimating equation (PROC GENMOD) as number of events per 5-h exposure day, including each rat’s baseline as its own intercept and assuming a Poisson distribution and exchangeable correlation structure. PROC REG was used to compare all BRS or arrhythmia values to physiologic parameters (simple linear regression) or pollutant concentrations (partial regression accounting for group).

## Results

### Diet effects

During baseline, MetS rats had increased BP, SDNN, and aortic dP/dt_max_ relative to control-diet rats but no difference in BRS slope (Table 1). Additional HRV analyses confirmed our initial observations (Additional file 1: Table S3 and Table S4). Eight-week HFrD corresponded with a 152-g increase in mean body mass (311 ± 5 to 463 ± 10 g), equivalent to body mass change in normal diet SD rats over the same time as previously noted [7]. At the conclusion of the study, air-exposed MetS rats had hyperglycemia (mean ± SEM: 243.8 ± 18.4 mg/dL) relative to a historic range for normal diet-fed male Sprague Dawley rats at a corresponding age of 20 weeks (50–135 mg/dL), with no additional effect of P + SOA.

**Table 1:** Baseline cardiovascular physiology in control-fed (*n* = 8) and MetS (*n* = 12) rats

	ND		MetS	
Systolic BP (mmHg)	121	± 1.9	*143.9*	± 5.8***
Diastolic BP (mmHg)	86.6	± 1.3	*95.7*	± 4.3*
*dP/dt*_*max*_ (mmHg/s)	1487	± 49	*2093*	± 105***
Heart Rate (beats/min)	333.3	± 5.1	328.6	± 5.8
BRS slope (ms/mmHg)	1.81	± 0.28	1.65	± 0.23
RMSSD (ms)	4.1	± 0.34	5.05	± 0.68
SDNN (ms)	6.85	± 0.47	*8.62*	± 0.50*
HF (ms^2^)^a^	1.37	± 0.22	2.55	± 0.73
LF (ms^2^)^a^	0.90	± 0.20	0.98	± 0.35
LF/HF†	0.64	± 0.05	0.50	± 0.06

Means ± SE from baseline FA exposure. ^a^indicates values from ECG + BP telemetered ND rats under the same baseline monitoring and FA conditions (*n* = 14). * and *** indicate *P* < 0.05 and <0.001, respectively

### P + SOA exposure

Overall, MetS-P + SOA rats received PM_2.5_ at concentrations 64% lower by mass and 27% lower by count than ND-P + SOA rats (Table 2 *).* Concentrations remained consistent throughout the entire exposure regimen (Additional file 1: Fig. S1). By count, approximately half of particulates were ultrafine for MetS-P + SOA rats and slightly fewer than half were ultrafine for ND-P + SOA rats. Additional information regarding P + SOA composition (organic carbon, elemental carbon, nitrate, and sulfate fractions) is presented in Additional file 1: Table S1.

**Table 2:** Characteristics of P + SOA exposure

Pollutant	ND		MetS	
PM_2.5_ Mass (μg/m^3^)	56.3	± 1.2	20.4	± 0.9
PM_2.5_ Count (thousand/cm^3^)	9.0	± 0.2	6.6	± 0.2
PM_2.5_ MMD (nm)	297.0	± 6	262.7	± 2
PM_2.5_ CMD (nm)	135.0	± 2	95.2	± 2
NO (ppb)	1.0	± 0	3.2	± 1.2
NO_x_ (ppb)	2.6	± 0.5	11.3	± 1
O_3_ (ppb)	-	-	22.7	± 2.1
CO (ppm)	-	-	1.0	±0

Means ± SE over entire 12-day exposure period. PM_2.5_ MMD and CMD denote mass and count median diameters ± geometric standard deviation. P + SOA exposure of ND rats applied the same parallel gas denuders as for MetS, but CO and O_3_ were not measured. FA was not monitored

### Autonomic balance

**Fig. 1:**
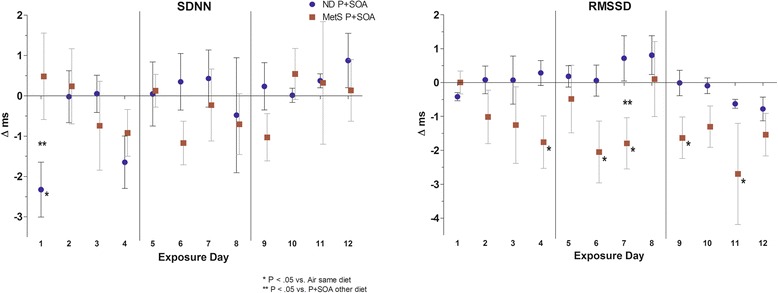
Daily time-domain HRV in ND and MetS rats. Values represent mean daily difference from diet-matched air-exposed group in change from baseline (±SE, *n* = 4–6/group) *—*P* < 0.05 vs. Air same diet. **—*P* < 0.05 vs. P + SOA other diet. Vertical lines indicate weekends

P + SOA significantly altered HRV in MetS rats, decreasing RMSSD delta by 1.31 ms overall (Table 3) and on nearly half of exposure days (Fig. 1) relative to MetS-FA rats. In contrast, P + SOA did not alter time domain HRV parameters in ND rats overall and only significantly decreased SDNN delta on day 1 relative to ND-FA rats.

**Table 3:** Overall Effects of P + SOA exposure on ND and MetS rats

		ND		MetS		MetS vs. ND
		*effect*	*P*	*effect*	*P*	*P*
	Diastolic Pressure (mmHg)	-0.45	0.874	−0.532	0.830	0.1073
	Systolic Pressure (mmHg)	2.97*	*0.044*	−1.26	0.692	0.021*
	Aortic *dP/dt*_*max*_ (mmHg/ms)	157*	*0.013*	−109*	*0.007*	<0.001*
	Pulse Pressure (mmHg)	3.26*	*0.020*	−1.97*	*0.035*	<0.001*
	Rate × Pressure (BPM*mmHg/1000)	0.098	0.927	−0.860	0.654	0.05*
	Heart Rate (BPM)	4.70	0.660	−1.74	0.814	0.458
	BRS (ms/mmHg)	0.167	0.807	−0.338	0.095	0.009*
HRV	RMSSD (ms)	−0.023	0.946	−1.31*	*0.027*	0.076
	SDNN (ms)	−0.101	0.855	−0.245	0.729	0.913
	HF (ms^2^)			−0.921	0.068	
	LF (ms^2^)			0.116	0.630	
ECG	PR Interval (ms)			−1.27*	*0.036*	
	QTc Interval (ms)			−2.16*	*0.025*	

Overall differences between diet-matched P + SOA- and FA-exposed groups in cardiovascular physiologic deltas from baseline or between opposing diets with exposure to P + SOA (right column, significance only). *indicates *P* < 0.05 by linear mixed model analysis

The italic font indicated significant differences

### Hemodynamics

**Fig. 2:**
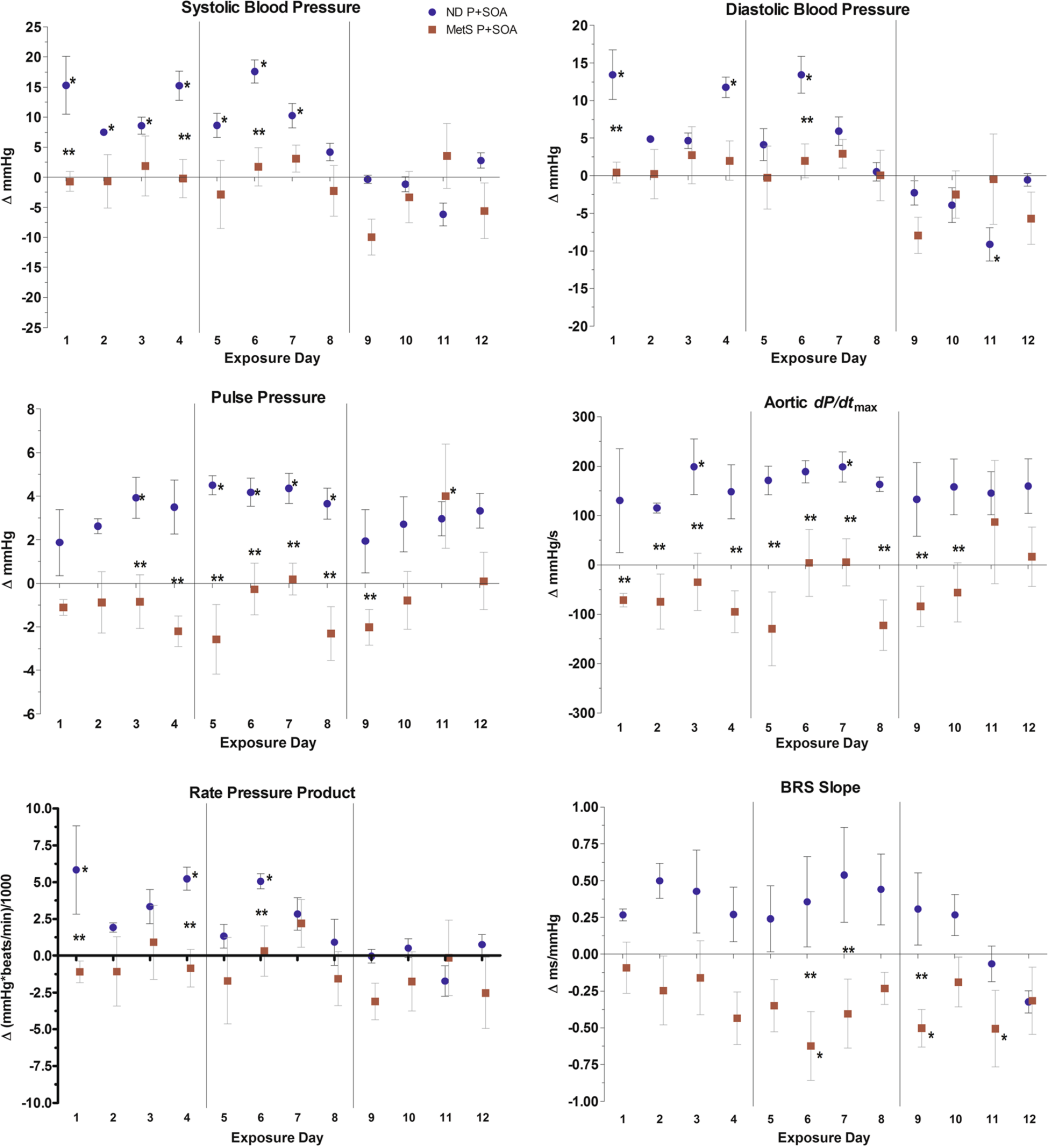
Hemodynamics in ND and MetS rats. Values represent mean daily difference from diet-matched air-exposed group in change from baseline (± SE, *n* = 4–6/group). ﻿﻿*—P < 0.05 vs. Air same diet. **—P < 0.05 vs. P + SOA other diet. Vertical lines indicate weekends.﻿

In ND rats, P + SOA overall significantly increased systolic pressure, aortic *dp/dt*_*max*_, and pulse pressure (PP) deltas vs. FA (Table 3). Conversely, P + SOA exposure in MetS rats decreased *dP/dt*_*max*_ and PP deltas overall vs. both the MetS-FA and ND-P + SOA groups, and P + SOA exposure decreased rate pressure product delta in MetS rats relative to P + SOA-exposed ND rats. P + SOA only affected MetS rats’ daily pressures on day 11 via an increase in PP (vs. MetS-FA), whereas it increased systolic, diastolic, and/or PP deltas among ND rats relative to the ND-FA group on the first eight exposure days but decreased diastolic pressure on day 11 (Fig. 2). On many of the exposure days, MetS-P + SOA rats also significantly diverged from ND-P + SOA rats in aortic *dP/dt*_*max*_ (days 1–10) and PP (days 3–9) deltas. BP did not associate with HRV in either strain (Table 4).

**Table 4:** Relationship between HRV and blood pressure changes from baseline in ND and MetS rats

		Δ SBP		Δ DBP		Δ MBP	
		*r*	*p*-value	*r*	*p*-value	*r*	*p*-value
ND rats	Δ RMSSD	0.05	0.63	0.15	0.16	0.11	0.31
	Δ SDNN	0.04	0.71	−0.08	0.48	−0.01	0.94
MetS rats	Δ RMSSD	-0.06	0.51	0.00	0.99	−0.02	0.83
	Δ SDNN	0.05	0.58	0.10	0.23	0.07	0.39

Pearson correlation coefficients and *P*-values for linear regression between HRV delta and various aortic pressure deltas among all MetS and ND rats during exposure periods

### Baroreflexes

MetS-P + SOA rats had decreased BRS slope delta vs. both MetS-FA rats and ND-P + SOA rats on three exposure days (Fig. 2). As well, MetS-P + SOA rats had decreased BRS slope delta relative to ND-P + SOA rats overall (−0.47 ms/mmHg, *P* < 0.01) and a trend of similar effect vs. MetS-FA rats (−0.34 ms/mmHg, *P* = 0.0946). Among all MetS rats, BRS slope delta correlated positively with HRV deltas and inversely with HR, PP, and *dP/dt*_*max*_ deltas (Table 5 *)*, all *P* < 0.0001) as well as rate-pressure product (*P* < 0.001). Similarly, in ND rats BRS slope delta positively correlated with CV and inversely correlated with PP and *dP/dt*_*max*_ deltas, consistent with autonomic modulation of HRV, cardiac inotropy, and baroreflexes.

**Table 5:** Correlation between BRS and physiologic markers, PM_2.5_ concentrations, or gaseous components

	ND	MetS
HR (beats/min)	0.23*	-0.28**
RMSSD (ms)	0.18	0.71***
SDNN (ms)	0.19	0.49***
CV	0.27**	0.40***
pNN15	-	0.59***
HF (ms^2^)	-	0.58***
LF (ms^2^)	-	0.71***
LF/HF	-	-0.13
SBP (mmHg)	0.00	-0.16
DBP (mmHg)	0.28**	−0.08
PP (mmHg)	−0.49***	−0.32***
*dP/dt*_*max*_ (mmHg/s)	−0.41***	−0.35***
rate × P (BPM × mmHg)	−0.06	-0.27**
PR (ms)	-	0.27**
QTcB (ms)	-	0.32***
QTcF (ms)	-	0.24**
QTe (ms)	-	−0.11
S min. Slope (mV × ms)	-	0.01
ST amp (mV)	-	0.13
TpTe (ms)	-	0.17*
*f* (breaths/min)	-	-0.41***
*T*_I_ (ms)	-	0.07
*T*_*E*_ (ms)	-	0.36***
*T*_*T*_ (ms)	-	0.35***
Pause (s)	-	0.14
PM_2.5_ mass (μg/m^3^)	−0.04	-0.11
PM_2.5_ count (#/m^3^)	−0.03	−0.05
NO (ppb)	0.00	−0.09
NO_x_ (ppb)	−0.01	−0.12
O_3_ (ppb)	-	0.03
CO (ppm)	-	−0.04

Pearson correlation coefficients for linear regression between BRS slope and various physiologic and exposure endpoints in MetS and ND-fed rats over the course of the entire exposure period, including both filtered air- and P + SOA-exposed animals. Pearson partial correlation was conducted for air pollutant concentrations. *, **, and *** indicate *P* < 0.05, 0.01, and 0.001, respectively. Dash alone indicates parameter was not analyzed

### Arrhythmia and ECG

**Fig. 3:**
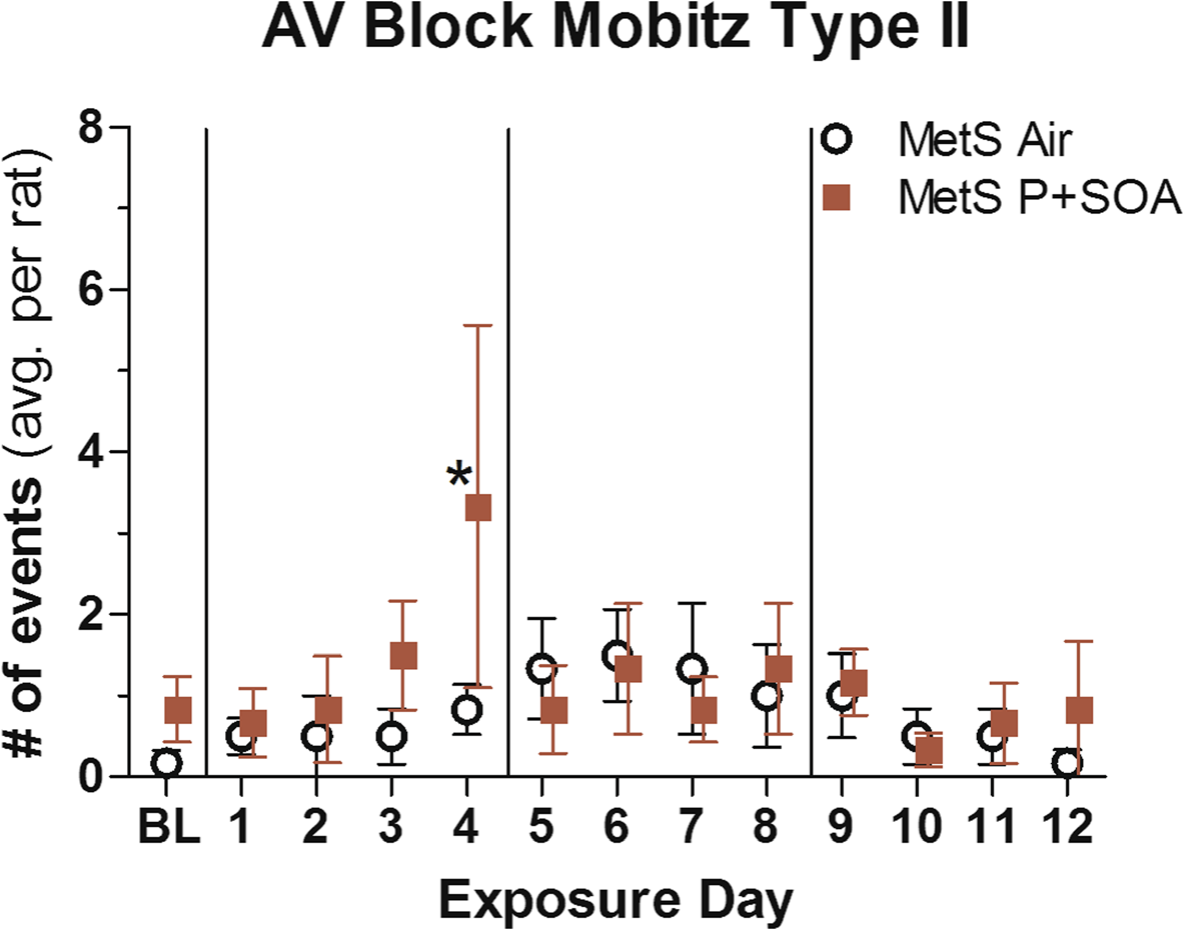
P + SOA exposure increased Atrioventricular Block Mobitz Type II arrhythmias in MetS rats on specific days relative to Air-control. Data are expressed as means ± SE (*N* = 6/group)

**Fig. 4:**
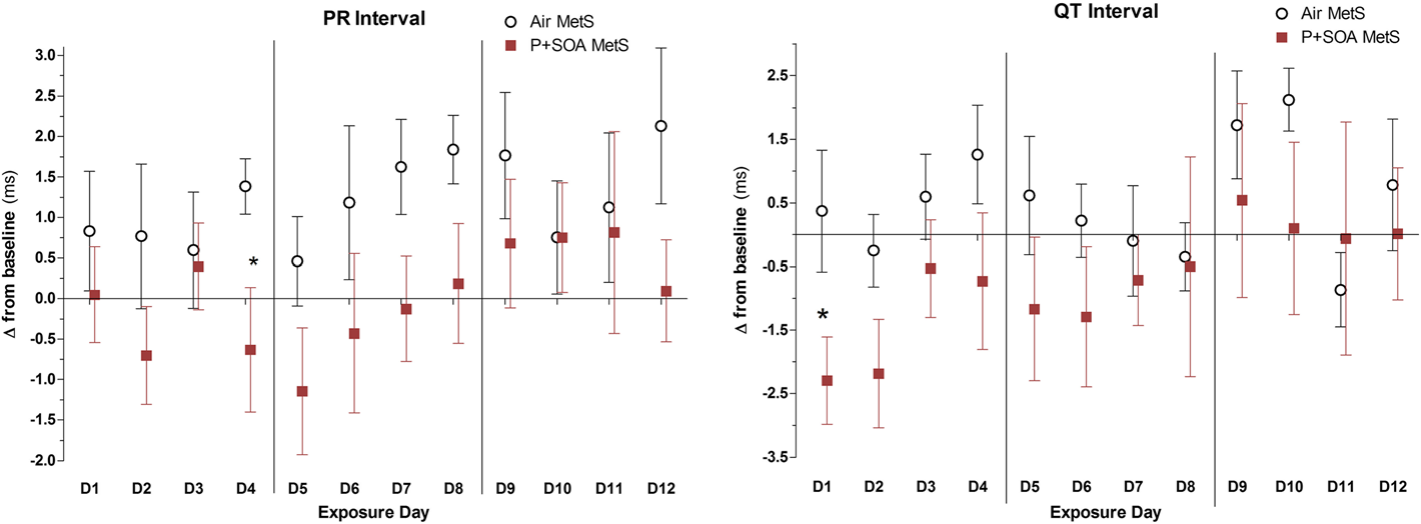
Daily change in ECG PR and QTc (Fridericia) interval from baseline in MetS rats during exposure to FA (Air) or P + SOA. Values represent group mean of daily change from individual rats’ values at baseline (±SE, *n* = 6/group). Vertical lines delineate exposure weeks. Asterisk denotes significant difference between time-matched group averages (*P* < 0.05)

On exposure day 4 there was a four-fold increase in second degree Atrioventricular (AV) block Mobitz type II arrhythmias in MetS-P + SOA rats compared to MetS-FA rats (Fig. 3), corresponding with a significant decline in PR interval delta (Fig. 4). These arrhythmias, which are relatively rare, occurred in half of the P + SOA-exposed rats on day 4. MetS-P + SOA rats had decreased QTc and PR interval deltas overall (Table 3 and Fig. 4). Interestingly, two ND rats exposed to P + SOA had paroxysmal tachycardia (HR > 500 beats/min) of undiscernible anatomical origin, with one sustained through day eight’s exposure and the other for twelve minutes on day 7. Among Mets rats, HRV, PR, and QTc inversely correlated with AV block Mobitz II arrhythmias, whereas these markers positively correlated with AV block Mobitz I, indicating potentially divergent autonomic origins (all *P* < 0.05; Additional file 1: Table S5*).* Second degree AV block Mobitz I events are a common benign consequence of parasympathetic activation-mediated vagal inhibition of the AV node, whereas Mobitz II events are a clinical indication for pacemaker implantation due to underlying pathology, including but not limited to potential inflammation and/or fibrosis.

### Ventilatory parameters

**Fig. 5:**
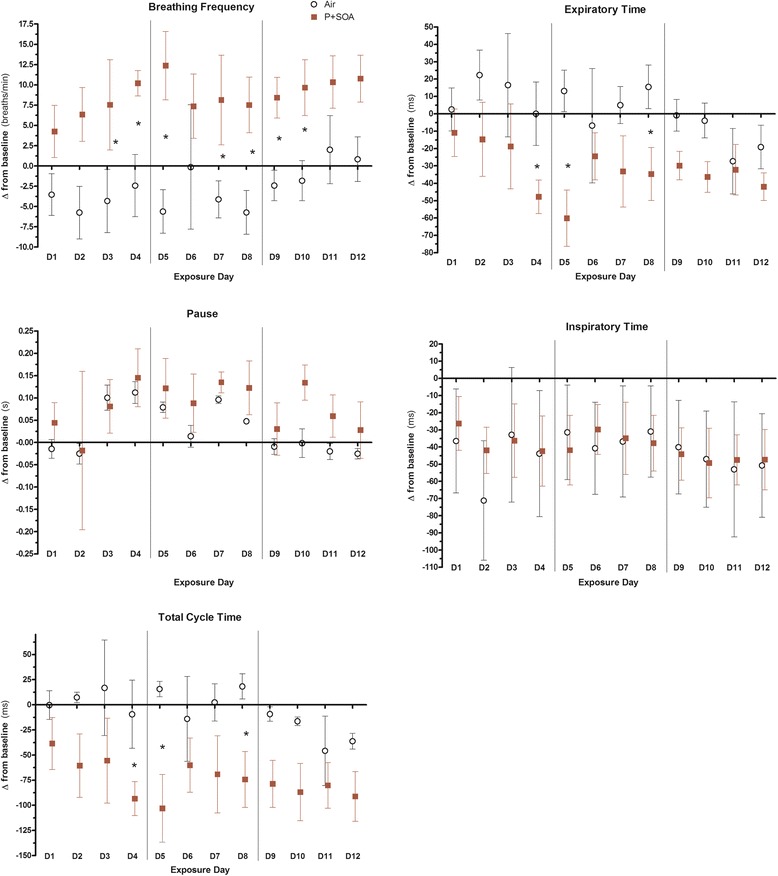
Daily change from baseline in respiratory function in MetS rats during exposure to FA (Air) or P + SOA. Values represent group mean of daily change from individual rats’ values at baseline (±SE, *n* = 5–6/group). Vertical lines delineate exposure weeks. Asterisk denotes significant difference between time-matched group averages (*P* < 0.05)

PM_2.5_ increased *f* by 11.2 breaths per minute and decreased T_E_ and T_T_ by 33.2 ms and 68.5 ms, respectively, in MetS rats overall and had similar effects on several individual days without affecting T_I_ or Pause (Fig. 5). Among MetS rats *f* and T_T_ deltas correlated with RMSSD and HF deltas, whereas T_I_ and Pause deltas correlated with SDNN delta (Additional file 1: Table S6).

### Predictors of arrhythmia

BRS slope inversely correlated with half of arrhythmia types, including AV block Mobitz type II, which itself also inversely correlated with most HRV parameters (including RMSSD), respiratory pause, PR, QTc, and ST amplitude (an index of myocardial ischemia) (Additional file 1: Table S5). In contrast, Mobitz type I AV block correlated positively with most of the aforementioned parameters, affirming its likely vagal origins and indicating divergent etiologies between Mobitz I and Mobitz II AV block events. Accordingly, HRV index of relative sympathetic influence (LF/HF) positively correlated with AV block Mobitz II events in MetS rats.

We examined respiratory flow waves of MetS rats on half of exposure days (days 1, 4, 6, 8, 11, and 12) for ventilatory rhythm disturbances (abrupt braking, apnea, or tachypnea) in the 3 s before arrhythmias to determine if these bradyarrhythmias immediately followed indications of vagal afferent-mediated irritant reflexes (characterized by tachypnea and respiratory braking with C-fiber activation [24]). Most Mobitz II events immediately followed ventilatory rhythm disturbances regardless of P + SOA or filtered air exposure; however, P + SOA exposure unexpectedly corresponded with two-fold *more* Mobitz II events preceded by undisturbed respiratory rhythm (36% vs. 19% of events relative to FA) and equivalent respiratory disturbance-associated Mobitz II events. When Mobitz II events significantly increased in P + SOA-exposed rats (day 4), they occurred even more disproportionately with normal respiratory waves (43%, vs. 9% in FA), accounting entirely for the group difference since ventilatory rhythm disturbance-associated events remained equal between groups.

## Discussion

We found in a HFrD-fed rat model of MetS that repeated traffic-derived PM_2.5_ exposure at environmentally relevant concentrations decreases relative vagal influence over cardiovascular function, diminishes baroreflexes to arterial pressure fluctuations, and alters electrophysiology concomitant with increased spontaneous arrhythmia. In contrast, normal diet rats showed minimal effects of PM_2.5_ on BRS and HRV while having consistent hypertensive responses in the first seven days of exposure that vanished thereafter. The declines in HRV and BRS and their association with increasing arrhythmia among MetS rats suggest repeat exposure to ambient PM_2.5_ impairs autonomic regulation of cardiovascular homeostasis especially among those with cardiometabolic disorder. P + SOA also diminished SDNN in normal-fed rats, but this occurred only on day 1 of exposure. The lack of recurrence of these effects in ND rats suggests that normal animals may acutely respond similarly to initial PM exposure but may thereafter mount compensatory adaptations to preempt subsequent effects. To our knowledge, this is the first demonstration that controlled exposure to traffic-derived PM_2.5_ at ambient levels impairs baroreflexes and causes spontaneous arrhythmia in conjunction with depressed HRV. Collectively, our observations indicate that MetS increases susceptibility to the adverse effects of ambient-level PM_2.5_, likely via ANS imbalance.

Overall, our findings suggest MetS increases cardiovascular susceptibility to PM_2.5_ via sympathetic dominance, indicated by decreases in RMSSD, spontaneous BRS, and PR interval. HRV indicates autonomic regulation of cardiac rhythm, and decreases in most HRV parameters (e.g., RMSSD, SDNN, HF) indicate increased sympathetic influence. Unlike healthy, ND-fed rats, HFrD-fed rats with MetS responded to PM_2.5_ exposure with sustained decreases in HRV and BRS, as well as tachypnea, and QTc- and PR-shortening, all of which are tied to sympathetic dominance [25–28]. In fact, we found significant correlations between HRV and tachypnea suggesting an important coupling between PM_2.5_-induced alterations in respiration and cardiac autonomic imbalance. RMSSD derives in part from respiratory sinus arrhythmia, involving the acceleration of pacemaker nodal rhythm via an abrupt parasympathetic withdrawal during stimulation of pulmonary stretch receptors [29]. As such, decreases in RMSSD may derive from (i) decreased stimulation of respiratory stretch receptors, (ii) sympathetic antagonism of parasympathetic-mediated respiratory sinus arrhythmia [30, 31], and/or (iii) impaired integrity of vagal efferent neurons [32].

In ND rats P + SOA exposure had notable hypertensive effects that we have previously discussed in detail [12]. The absence of a hypertensive effect of P + SOA exposure in MetS rats may stem from a number of factors, including dose dependency of P + SOA. MetS rats were exposed to lower P + SOA concentrations than the ND rats, having average PM_2.5_ mass and count concentrations that were 36% and 73% of those that the ND rats received. As well, rats fed a normal diet and having normal blood pressure may have had greater potential for hypertensive responses to PM_2.5_ simply because the MetS rats already had frank hypertension. Indeed, dietary fructose may have overshadowed any such hypertensive effects of PM_2.5_ in MetS rats through a common non-autonomic pathway (e.g., renin-angiotensin system activation). Others have revealed evidence that both PM_2.5_ and high fructose diets induce hypertension through the renin-angiotensin system [33, 34]. Similarly suggesting our hemodynamic observations were unrelated to autonomic influences, we observed no statistical associations between changes in HRV and BP in either ND or MetS rats (Table 4). Accordingly, BRS slope also suggested PM_2.5_ uniquely caused autonomic-associated dysfunction only in MetS rats. The cardiac baroreflex is a negative feedback loop by which arterial pressure receptors relay BP changes to the brain, which inversely tunes heart rate by adjusting parasympathetic tone. Diminished baroreflexes indicate impaired compensatory responses to periodic increases in aortic pressure (and cardiac afterload). Ultimately, this may increase overall cardiac systolic workload and promote heart failure. Our laboratory previously observed in tracheostomized dogs that repeated exposures to much higher PM_2.5_ levels (358 μg/m^3^) increase BP and BRS, likely in compensation for PM_2.5_’s hypertensive effects [35]. Although PM_2.5_ did not significantly increase BRS in ND rats despite its hypertensive effects, differences in species and exposure traits hinder comparability with this past study. Nevertheless, the decreases in BRS and HRV suggest MetS increases susceptibility to PM_2.5_-induced cardiovascular dysfunction via the ANS.

Our observations lend further plausibility to the mounting evidence that PM_2.5_ causes adverse cardiac outcomes through sympathetic dominance [1]. Such effects have been tied to autonomic imbalance and found to either herald or precipitate cardiovascular mortality. La Rovere and colleagues [28] found that depressions in BRS and HRV independently predicted long-term mortality and synergistically foretold a seven-fold increase in mortality rates among myocardial infarction survivors. Spontaneous BRS has also been inversely correlated with mortality and adverse cardiovascular events [23]. As well, low aortic dP/dt_max_—an index of left ventricular contractility—has been deemed predictive of death and transplantation in heart failure patients [21]. Separately, Mobitz type II AV block arrhythmia often progresses to complete heart block in humans [36] and is considered a high-grade bradyarrhythmia, which others have attributed to one sixth of ambulatory sudden cardiac deaths [37]. Likewise, QT and PR shortening are both established predictors of mortality [38, 39]. Although others have made similar observations to ours, they have typically involved air pollutants far exceeding ambient concentrations. Hazari and colleagues [40] recently reported acute inhalation of the combustion product acrolein at levels >1000-fold [41] traffic-congested urban environments causes strikingly similar effects on BRS, AV block arrhythmia, QT, PR, and HRV. Conversely, we saw effects in MetS rats at PM_2.5_ levels with a 24-h average < 1/8th the daily U.S. Environmental Protection Agency standard (4.3 vs. 35 μg/m^3^).

**Fig. 6:**
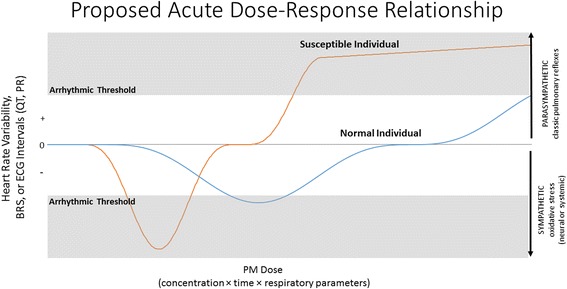
Proposed relationship between cardiac physiology and acute particulate exposure based on susceptibility (e.g., hypertension, MetS, age, heart failure). With increasing concentration and/or exposure time, parasympathetic reflexes may counteract sympathetic dominance, dependent on antioxidant status, pulmonary clearance, vagal integrity, additional gaseous pollutants, and particulate interaction with irritant receptors

Short-term ambient PM_2.5_ exposures have been linked to greater HRV decrements in MetS subjects than in healthy subjects [4] or type 2 diabetics [42]. Yet, controlled exposures (involving five- to ten-fold higher concentrations) provide little evidence that MetS increases autonomic or hemodynamic susceptibility [43, 44]. Using the same fructose-induced MetS rat model as ours, others found that a 9-day CAPs exposure at higher concentrations (356 μg/m^3^) enhanced oxidative stress and inflammation in epicardial adipose tissue [7] but also increased HRV divergent from normal diet rats [6]. Indeed, acute exposure to similarly high PM_2.5_ concentrations has repeatedly elicited transient parasympathetic dominance in rodent models of cardiovascular disease [45–47]. Such vagal dominance likely stems from pulmonary and airway irritant reflexes that directly inhibit sympathetic neurons, manifesting as increased HRV at high pollutant levels in humans as well [48–50]. Fittingly, Farraj and colleagues [45] recently noted repeated exposure to biodiesel emissions in hypertensive rats decreased HRV at 50 μg/m^3^, had no effect at 150 μg/m^3^, and increased HRV at 500 μg/m^3^. Conversely, central oxidative stress can induce sympathetic dominance [51], and exogenous antioxidants may inhibit PM_2.5_-induced alterations in HRV [52]. Among MetS individuals exposed to PM_2.5_ (98 μg/m^3^), only those with compromised antioxidant defenses had decreased HRV [44]. Controlled exposures to lower PM_2.5_ levels (24–50 μg/m^3^) in diabetic, elderly, overweight, or coronary heart disease subjects can elicit similar decreases in HRV [53–56]. Exposures to ambient ultrafine PM (UFP: 10–100 nm diameter) and accumulation mode PM (AMP: 100–500 nm diameter) at levels comparable to our study have also been associated with decreased HRV and impaired ventricular repolarization in cardiac rehabilitation patients [57]. Yet the HRV-PM exposure relationship also can invert with beta-adrenergic blockade [56], suggesting that sympathoexcitation may obscure vagal pulmonary irritant reflexes at lower concentrations. Thus, separate dose-dependencies for these two competing autonomic pathways may drive a quasi-sinusoidal relationship between acute exposure and cardiophysiologic responses, varying with susceptibility (e.g., antioxidant status), respiratory deposition site, and particulate size and composition (Fig. 6). In this context, our findings indicate future controlled exposure studies may better elucidate the pathophysiologic effects of PM_2.5_ through multi-day exposures at real-world levels.

Increased sympathetic tone to the heart promotes arrhythmia, oxidative stress, contractile dysfunction, and eventual cardiac remodeling via distinct molecular pathways [58–60]. Nevertheless, acute sympathetic dominance only alludes to cardiac pathogenesis. In contrast, AV block Mobitz type II arrhythmia is a tangible marker of cardiac instability and a clinical indication for pacemaker implantation. Additionally, HRV and BRS inversely correlate with risk for fatal arrhythmia [28]. Indeed, many epidemiological studies suggest ambient PM_2.5_ exposure decreases HRV [1] and causes spontaneous arrhythmia and cardiac arrest [61]. Although sympathetic dominance likely drives PM_2.5_-induced cardiac arrhythmia and arrest, human studies have yet to confirm this link. In contrast, Hazari [62] demonstrated in hypertensive rats that diesel exhaust inhalation enhances sensitivity to drug-induced arrhythmia and cardiac arrest via a sympathetic pathway. Herein, we eschewed additional arrhythmogenic triggers, likely explaining the transience of our observations. Dose-dependent trends for Mobitz II AV block and HRV through the first exposure week culminated in significant changes on day 4, yet no similar cumulative effects appeared over the following two weeks, perhaps due to compensatory responses. The correlation of these arrhythmias with HRV, BRS, and ECG intervals further suggests their sympathetic etiology, but additional studies are needed to confirm this link. In contrast, the more benign Mobitz I AV block correlated with these parameters in the opposite direction, suggesting these arrhythmia types diverge in etiology and deserve careful distinction in subsequent studies.

It remains unclear whether pollutant exposures might precipitate sudden cardiac death by provoking tachy- or bradyarrythmias, but PM_2.5_ exposure appears to disproportionately associate with non-fatal tachyarrhythmias in humans. PM_2.5_ induces tachyarrhythmias almost exclusively in rodent models of surgically-induced myocardial infarction [63], whereas rodent models of hypertension or progressive cardiomyopathy repeatedly respond to PM_2.5_ with bradyarrhythmias resembling AV block Mobitz type II. These discrepancies may stem from inherent differences in cardiac phenotype between humans and rodents. Notably, in Mets rats PM-induced AV block Mobitz II events were inversely related to ventilatory pause and RMSSD, and their increase was entirely accounted for by AV block events uncoupled from acute respiratory disruptions that derive from vagal activation [24]. Ventilatory disturbances (spontaneous braking, tachypnea, or apnea) may initiate compensatory autonomic reflexes to resynchronize cardioventilatory rhythm [64], and abrupt increases in parasympathetic modulation can cause AV block Mobitz II events [65–67]. Mobitz II events inversely correlated with daily averages of RMSSD and PR interval (derived from 5-min intervals), suggesting a positive correlation in MetS rats between sympathetic dominance and these arrhythmias. Ostensibly, these Mobitz II events may have occurred via acute parasympathetic compensation to tonic elevations in sympathetic influence induced by PM. Although we did not test for acute shifts in autonomic balance, others have noted that concurrent increases in sympathetic and parasympathetic tone, or the transition from parasympathetic excitation to a sympathetic surge, may induce atrial and ventricular fibrillation [68].

AV block Mobitz II arrhythmias are considered arrhythmias with sufficient potential for full AV dissociation, complete heart block, and cardiac arrest such that they are a clinical indication for pacemaker implantation [69]. Repeat exposure to ten-fold higher PM_2.5_ levels has been shown to impair cardiac vagal neuron excitability [32], which in turn may shorten PR interval, impair AV conduction, and thus promote Mobitz II AV block arrhythmias. Finally, the PM_2.5_-induced QT shortening in MetS rats recapitulates findings in humans [70] and denotes an increased risk for arrhythmia and sudden cardiac death further corroborated by its correlations with arrhythmia. Ultimately, our observations indicate MetS increases susceptibility to PM_2.5_-induced autonomic imbalance with associated arrhythmia, but further studies are required to unveil the basis of this susceptibility.

### Strengths and limitations

Photochemical reaction of the traffic aerosol with gaseous pollutants enhanced ecological validity by simulating ambient generation of SOA at a consistent rate, whereas denuders ensured gases were within environmental norms. Nevertheless, the different PM_2.5_ concentrations between P + SOA-exposed ND and MetS rats hinders statistical determination of whether a phenotype-aerosol interaction accounted for the effects in MetS-P + SOA rats. Moreover, the cause of differences in aerosol concentrations remains unclear, particularly as we do not have data characterizing vehicular traffic or PM composition. Although the reasons remain unclear, P + SOA concentrations may have been lower for MetS rats due to diminished SOA production in the photoreaction chamber secondary to a higher primary PM concentration in the tunnel plenum, as primary particles act as sinks for radicals and thus prevent the formation of SOA. Concordant with this, the elemental carbon (EC) fraction was notably higher for the MetS aerosol exposure. Nevertheless, EC was low for environmental levels, indicating vehicular particles accounted for only a small portion of the P + SOA mass for ND and MetS rats. Although MetS rats had greater physiologic responses at less than half the PM_2.5_ concentration of ND rats, it is also possible a higher dose might elicit blunted responses per our hypothesized dose-response relationship. Consequently, inferences from this study should be tempered by the limited comparability between aerosol exposure concentrations for the MetS and ND rats. It should also be noted that roughly half of the P + SOA particles were in the ultrafine range (< 100 nm). In urban airsheds of post-industrial nations, background ultrafine PM (UFP) concentrations have been recently observed at comparable or higher levels [57, 71, 72]. Nevertheless, comparisons of our findings to other studies primarily incorporating larger particles might be hindered by divergent kinetics due to a lower deposition fraction in the bronchioles and parenchyma and differential mucociliary clearance and inflammatory cell interactions. The enhanced deposition of UFP may also augment activation of irritant receptors and thus autonomic effects relative to AMP or larger particles. Contrary to this, interquartile increases in ambient AMP were followed by more overt impairments in RMSSD and ventricular repolarization in cardiac rehabilitation patients than interquartile increases in UFP [57].

MetS rats had higher HRV than ND rats prior to PM_2.5_ exposure. Although somewhat unconventional, these findings are consistent with daytime measures in high fructose-fed mice [34] and obese children [73], and may derive from unique dietary-induced features of the model such as baroreflexes involving parasympathetic compensation for fructose-induced hypertension. This seems particularly plausible given that baroreflex sensitivity was unaffected by fructose alone; however, we were unable to assess systolic pressure variability, which may have informed whether greater volatility in aortic pressure corresponded with increased parasympathetic modulation in MetS rats. We analyzed HRV using time domain, power domain, and non-linear parameters in 5-min segments sampled continuously from waveforms while controlling for confounding arrhythmias and artifacts to provide an assessment generally more rigorous and comprehensive than conventional approaches (analysis for 30 s of every 5 min [6]; 4 min of every 10 min [46]; 60 s of every 5 min [45]).

It should be noted that the hypertension and hyperglycemia in these MetS rats recapitulates effects in the same strain, gender, and age fed the same formula for the same duration [6], which also induced insulin resistance (increased HOMA-IR) and hypertriglyceridemia. Concurrent with the hypertension and hyperglycemia that we observed, MetS rats also likely had similar insulin resistance and hypertriglyceridemia. Although we did not verify hypertriglyceridemia and insulin resistance in the present study, it is apparent that our model collectively satisfies the criteria for MetS.

Although we did not pharmacologically validate our assessment of spontaneous BRS slope here, the sequence method has been validated relative to conventional clinical assessments of BRS [23]. Furthermore, we recently observed decreases in both spontaneous and nitroprusside-derived BRS slope in a follow-up pilot with highway traffic tunnel PM-exposed rats (unpublished data). The discordance from our prior observations of enhanced BRS in PM-exposed tracheostomized dogs may derive from this prior exposure bypassing upper airway (e.g., nasal) irritant receptors, or it may derive from key differences between the rat model of MetS and otherwise normal canines. The lack of ECG in ND rats preclude any comparison of PM_2.5_-induced arrhythmia with MetS rats. Nevertheless, PM_2.5_-exposed MetS rats had disproportionate and concomitant depressions in HRV and BRS, which have been shown to more robustly predict cardiovascular mortality than non-sustained ventricular tachycardia [28]. Finally, our findings are relatively descriptive. While we believe that the confluence of physiologic effects of PM on Mets rats (RMSSD decline, BRS decline, PR shortening, QT shortening, and increased AV block Mobitz II arrhythmia) and their correlation with each other collectively suggest that MetS increases cardiovascular susceptibility to environmentally relevant levels of PM via decreases in parasympathetic modulation, confirmation of autonomic mediation requires more sophisticated studies involving molecular or neural interventions.

## Conclusions

Traffic-derived PM_2.5_ exposure at ambient levels blunts parasympathetic influence over cardiovascular function, induces tachypnea, and alters cardiac repolarization while transiently increasing spontaneous arrhythmia in a rat model of MetS. Individuals with MetS may be more susceptible to the adverse cardiac effects of PM_2.5_ exposure, and exposure to traffic-derived PM_2.5_ at concentrations common in the U.S. may measurably harm cardiovascular health through dysfunction of the autonomic nervous system.

## Additional file

Additional file 1: Supplemental Data, **Tables S1**-**S6** and **Figures S1**-**S3**. (DOCX 94 kb)

## Abbreviations

ANS: autonomic nervous system
APB: atrial premature beat
AV: atrioventricular
BP: blood pressure
BPM: beats per minute
BRS: baroreflex sensitivity
CAPs: concentrated ambient particulates
CMD: count median diameter
CO: carbon monoxide
CPC: condensation particle counter
CV: coefficient of variation
dP/dt_max_: maximum rate of increase in aortic pressure per beat
ECG: electrocardiogram
F: respiratory frequency
FA: filtered air
HF: high frequency power spectral HRV
HFrD: high fructose diet
HRV: heart rate variability
LF: low frequency power spectral HRV
MetS: metabolic syndrome
MMD: mass median diameter
Ms.: milliseconds
ND: normal diet
NO: nitric oxide
NO_x_: nitrogen oxides
O_3_: ozone
P + SOA: primary and secondary organic aerosols
PM_2.5_: particulate matter <2.5 um in diameter
PP: pulse pressure
RMSSD: root mean squared of successive differences
SD: Sprague Dawley
SDNN: standard deviation of normal interbeat intervals
SE: standard error
SMPS: scanning mobility particle sizer
TE: expiratory time
TI: inspiratory time
TT: total respiratory cycle time
