# Supplementary material for: Inhaled ambient-level traffic-derived particulates decrease cardiac vagal influence and baroreflexes and increase arrhythmia in a rat model of metabolic syndrome
Source: Part Fibre Toxicol. 2017 May 25;14:16. doi: 10.1186/s12989-017-0196-2 (PMC5445437; doi:10.1186/s12989-017-0196-2)
Supplement: Additional file 1: — Supplemental Data, Tables S1-S6 and Figures S1-S3. (DOCX 94 kb) [file 12989_2017_196_MOESM1_ESM.docx]

**Supplemental Figure 1.** PM_2.5_ concentrations by mass and count during inhalation exposure of MetS and ND rats.

*P+SOA elemental carbon, organic carbon, nitrate, and sulfate analyses.*Aerosol samples were collected on quartz filters in two day increments over the entire exposure period, resulting in six total filter samples per study. Analyses were performed by the Environmental Chemistry Laboratory at the Harvard T. H. Chan School of Public Health, using thermal-optical reflectance (TOR) analysis. Particulate elemental carbon (EC) and organic carbon (OC) and their thermally-resolved fractions (EC1, EC2, EC3, OC1, OC2, OC3, and OC4) are operationally defined by TOR analysis (Chow et al., 1993) using the IMPROVE (Interagency Monitoring of Protected Visual Environments) carbon thermal evolution program. These fractions are defined based on the temperatures at which they evolve under two phases of gradual heating during TOR analysis. The first phase is an oxygen (O_2_) free, helium (He) environment and the second is a 2% O_2_/98% He environment [1]. Ideally, volatile and semi-volatile OC will evolve in the first phase by thermal desorption and EC will evolve in the second phase by oxidation. With the first four OC fractions, OC1 is generally composed of the most volatile organics, including some gas phase organic compounds adsorbed by the quartz fiber filter during sampling. OC2, OC3, and OC4 are evolved under progressively increasing temperatures, and are less volatile organic components of increasing molecular weight and/or polarity. During this first heating phase, some of the OC on the filter will pyrolyze or char, and subsequently evolve with EC by oxidation in the second phase of the analysis. This is determined by monitoring the laser reflectance of the filter. When char is forming, the filter darkens and the laser reflectance reading decreases. As the pyrolyzed OC (OP) is evolving, the laser reflectance reading increases until it reaches its initial level. This cut-point defines the OP fraction which is calculated using an optical correction method. During the second phase of the analysis, EC is evolved under oxidation and further increasing temperature. Particle nitrate and sulfate were analyzed by ion chromatography.

|  |  | **ND** | **MetS** |
| --- | --- | --- | --- |
| **OC** | **μg/m^3^** | 15.723 ± 1.451 | 6.913 ± 0.444 |
| **EC** | **μg/m^3^** | 0.621 ± 0.117 | 1.815 ± 0.150 |
| **TC** | **μg/m^3^** | 16.344 ± 1.541 | 8.730 ± 0.471 |
| **OC1** | **μg/m^3^** | 0.305 ± 0.023 | 0.369 ± 0.067 |
| **OC2** | **μg/m^3^** | 5.679 ± 0.275 | 2.176 ± 0.157 |
| **OC3** | **μg/m^3^** | 5.482 ± 0.639 | 2.511 ± 0.159 |
| **OC4** | **μg/m^3^** | 1.920 ± 0.245 | 0.939 ± 0.063 |
| **Pyrolized OC** | **μg/m^3^** | 2.336 ± 0.316 | 0.919 ± 0.103 |
| **Nitrate** | **μg/m^3^** | 1.579 ± 0.248 | 3.719 ± 0.414 |
| **Sulfate** | **μg/m^3^** | 3.187 ± 1.237 | 1.479 ± 0.364 |

**Supplemental Table 1.** Carbon, nitrate, and sulfate fractions by mass of aerosols delivered to ND and MetS rats, respectively.

For the P+SOA aerosol, organic carbon accounted for approximately 28% of the PM mass for ND rats and 31% of PM mass for MetS rats, with remaining mass mostly accounted for by additional atoms bound to carbon in organic molecules, as well as nitrate and sulfate. Per the photochemical generation of SOA, organics accounted for the bulk of the mass, whereas primary particles (accounting for the vast majority of EC) contributed only a small fraction of the total aerosol mass. The low elemental content and the need for most of the flow output to be delivered to animals rendered an insufficient mass loading to filters for trace elemental analysis. Because primary particles provide a sink for radicals that prevents the formation of SOA, dilution of tunnel aerosol was required before photoreaction such that the inflow to the photoreaction chamber involved 30% tunnel plenum air and 70% HEPA-filtered clean air. A higher PM concentration in the tunnel plenum may have accounted for the higher EC values in MetS exposure and resulted in a lower production of SOA and overall mass. It should be noted that these EC concentrations for both aerosols are particularly low relative to ambient airsheds. Notably, the OC1 fraction, which represents the most volatile of PM- and filter-bound organics, were relatively comparable between exposures to Mets and ND rats.

|  | *r* | C.I. | p-value |
| --- | --- | --- | --- |
| Δ RMSSD (ms) | 0.80 | (0.62, 0.90) | <0.0001 |
| Δ SDNN (ms) | 0.85 | (0.71, 0.92) | <0.0001 |

**Supplemental Table 2.** Pearson correlation (r) and 95% confidence intervals (C.I.) for ECG- and BP-derived HRV changes from baseline for all MetS rats on representative exposure days (1, 6, and 11). Each rat’s ECG and BP waveforms were collected continuously through exposure, analyzed for HRV every sequential 5 min, and averaged into hourly and daily values. The correlations confirm that BP-derived HRV closely reflects ECG-derived HRV and support the use of BP waveforms for HRV analyses.

ND (n=14) MetS (n=12)

**
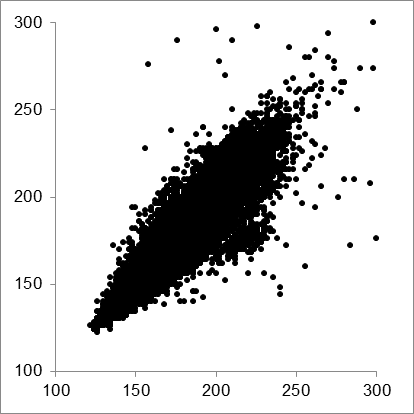
**
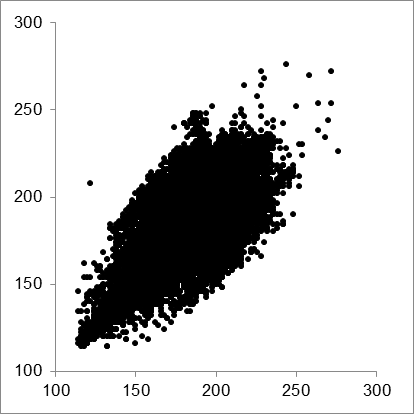


RR_n+1_ (ms)

RR_n+1_ (ms)

RR_n_ (ms)

RR_n_ (ms)

**Supplemental Figure 2.** Poincare plots of all rats’ filtered successive RR interval pairs during baseline (before PM exposure). The wider distribution among MetS rats is consistent with higher HRV resulting from an 8-week HFrD.

| ***HRV Index*** | ***ND (n=14)*** | ***MetS (n=12)*** |
| --- | --- | --- |
| **RR(ms)** | 178.8 ± 10.7 | 183.5 ± 10.7 |
| **HR(bpm)** | 338.2 ± 20.4 | 329.9 ± 19.9 |
| **SDNN (ms)** | 11.21 ± 2.46 | 13.07 ± 3.37 |
| **rMSSD (ms)** | **3.62 ± 1.06** | **5.24 ± 2.62*** |
| **TINN(ms)** | 91.1 ± 55.7 | 72.9 ± 15.7 |
| **RRtri** | 3.19 ± 0.76 | 3.55 ± 0.87 |
| **SD1(ms)** | **2.56 ± 0.76** | **3.70 ± 1.85*** |
| **SD2(ms)** | 15.6 ± 3.5 | 18.0 ± 4.8 |
| **SD1/SD2(ms)** | 0.17 ± 0.06 | 0.21 ± 0.10 |
| **LF (ms²)** | 9.50 ± 3.70 | 11.92 ± 4.76 |
| **HF (ms²)** | 2.14 ± 1.66 | 2.58 ± 2.54 |
| **LF (nu)** | 82.0 ± 6.6 | 83.5 ± 6.8 |
| **HF (nu)** | 18.0 ± 6.6 | 16.5 ± 6.8 |
| **LF/HF** | 5.29 ± 2.28 | 5.82 ± 2.21 |
| **Medialine** | 38.9 ± 9.3 | 36.4 ± 10.7 |
| **Maxline** | 673.1 ± 120.4 | 633.6 ± 186.1 |
| **RecurrenceRate** | 51.4 ± 3.4 | 48.9 ± 6.5 |
| **Determinism** | 99.2 ± 0.2 | 98.9 ± 0.7 |
| **Shannon Entropy** | 4.2 ± 0.2 | 4.1 ± 0.3 |
| **ApEn** | **1.19 ± 0.09** | **1.32 ± 0.21*** |
| **SampEn** | **1.18 ± 0.34** | **1.40 ± 0.20*** |
| **DC** | 0.0131 ± 0.0125 | 0.0229 ± 0.0192 |
| **Alfa 1** | 0.81 ± 0.08 | 0.75 ± 0.15 |
| **Alfa 2** | 1.36 ± 0.10 | 1.33 ± 0.11 |
| **Alfa1/Alfa 2** | 0.55 ± 0.09 | 0.57 ± 0.11 |

**Supplemental Table 3.** Mean (± standard deviation) of HRV during baseline exposure to FA. Significance (P < 0.05) was calculated from student’s independent t-tests except for italicized p-values, denoting Mann-Whitney test. Parameters include those derived from linear analysis (SDNN, RMSSD, TINN [triangular interpolation of RR]], and RRtri [triangular index]), Poincare measurements (SD1 and SD2 [standard deviation of instant and long-term beat-to-beat variabilities, respectively]); power spectral analysis (LF and HF in ms^2^ and normalized units); recurrence analysis (medialine, maxline, recurrence rate, determinism); entropy analysis (Shannon entropy, ApEn [Approximate entropy], SampEn [Sample entropy]; and fractal dimension (Alfa1, and Alfa2 and DC [dimension of correlation]). Analyses were performed in Kubios (version 2.0, Kuopio, Finland).

*Chaotic Global and Higuchi Fractal Dimension Analyses of Baseline HRV*. For non-linear analyses at Oxford Brookes University, 3200 of each rat’s interbeat intervals from baseline were filtered of artifacts and arrhythmias as described above. High spectral chaotic global techniques and spectral Multi-Taper Method (sMTM) were used blind to initial HRV results obtained at Harvard. After converting data to a power spectrum, a chaotic algorithm was applied for three chaotic global techniques with seven permutations termed Chaos Forward Parameters (CFP) as previously described [2, 3]. Data were converted to the multi-taper method power spectrum with standard parameters [3] yielding two high spectral versions. Anderson-Darling and Lilliefors tests [4] confirmed data normality. Principal Component Analysis indicated greatest variance within the first two components. MetS increased CFPs 1 and 3 relative to ND (Supplemental Table 3). CFP1 applies all three chaotic globals [3], whereas CFP3 lacks a high spectral detrended fluctuation analysis.

**Supplemental Table 4:** Means ± SE and P-values for CFPs 1-7 using unbalanced ANOVA1.

| Chaotic Globals Combination  [CFP 1-7] | ND | MetS | P |
| --- | --- | --- | --- |
| CFP1 | 1.0898 ±0.0116 | 1.1664 ±0.0102 | 0.0001 |
| CFP2 | 0.7045 ±0.0394 | 0.7412 ±0.0393 | 0.5181 |
| CFP3 | 1.0680 ±0.0133 | 1.1526 ±0.0122 | 0.0001 |
| CFP4 | 0.8390 ±0.0346 | 0.9044 ±0.0284 | 0.1651 |
| CFP5 | 0.1881 ±0.0293 | 0.1571 ±0.0251 | 0.4385 |
| CFP6 | 0.8135 ±0.0292 | 0.8879 ±0.0251 | 0.0701 |
| CFP7 | 0.6621 ±0.0492 | 0.7161 ±0.0446 | 0.4308 |

Higuchi Fractal Dimension [5] was separately applied without any prior spectral processing. Although MetS rats consistently exceeded ND rats at all fifteen K_max_ values, there were no significant differences, in contrast with the combinations of chaotic globals in CFP1 and CFP3. Thus, two combinations of high spectral chaotic global techniques and sMTM indicated greater interbeat interval complexity in MetS vs. ND at inhalation exposure baseline (p<0.0001). Obese children have shown similar elevations in chaotic global HRV relative to normal BMI children [6].

|  | **AVB Mobitz I** | **AVB Mobitz II** | **Adv. AVB** | **SAB** | **ncAPB** | **APB** | **A-tach** | **VPB** | **V-tach** |
| --- | --- | --- | --- | --- | --- | --- | --- | --- | --- |
| HR | -0.05 | -0.01 | 0.17* | 0.14 | 0.15 | -0.17 | -0.04 | 0.22** | 0.09 |
| RMSSD | 0.20* | -0.24** | -0.16 | -0.14 | -0.19 | 0.02 | -0.09 | -0.14 | 0.09 |
| SDNN | 0.29*** | -0.18* | -0.11 | 0.02 | 0.07 | 0.23 | 0.05 | 0.03 | 0.19* |
| pNN15 | 0.26** | -0.09 | -0.09 | -0.07 | -0.07 | 0.16 | -0.06 | -0.11 | 0.07 |
| HF | 0.09 | -0.18* | -0.13 | -0.07 | -0.10 | 0.04 | -0.06 | -0.08 | 0.06 |
| LF | 0.05 | -0.22** | -0.07 | 0.00 | -0.15 | 0.03 | 0.06 | 0.03 | 0.11 |
| LF/HF | 0.22** | 0.11 | 0.09 | 0.21** | 0.25* | 0.05 | 0.20* | 0.17* | 0.07 |
| SBP | -0.03 | 0.12 | 0.14 | -0.11 | 0.08 | 0.05 | -0.03 | 0.16 | 0.15 |
| DBP | 0.03 | 0.10 | 0.15 | -0.10 | 0.05 | 0.04 | -0.02 | 0.12 | 0.13 |
| PP | -0.18* | 0.12 | 0.04 | -0.09 | 0.12 | 0.08 | -0.04 | 0.19 | 0.11 |
| BRS slope | 0.12 | -0.25** | -0.28*** | -0.32*** | -0.40*** | -0.05 | -0.21* | -0.23** | 0.12 |
| RPP | -0.08 | 0.08 | 0.17* | 0.04 | 0.15 | -0.07 | -0.02 | 0.26** | 0.13 |
| PR | 0.23** | -0.22** | 0.03 | 0.10 | -0.03 | -0.11 | -0.03 | 0.07 | 0.14 |
| QTcB | 0.24** | -0.22** | 0.01 | 0.08 | -0.05 | -0.08 | -0.02 | 0.04 | 0.13 |
| QTcF | 0.24** | -0.30*** | -0.11 | 0.03 | -0.23 | 0.04 | 0.03 | 0.04 | -0.07 |
| QTe | 0.19* | 0.04 | -0.17* | -0.10 | -0.20 | -0.02 | -0.23** | -0.09 | -0.09 |
| S min. slope | 0.18* | 0.08 | -0.14 | -0.11 | -0.19 | 0.01 | -0.23** | -0.11 | -0.08 |
| ST amp | 0.11 | -0.28** | -0.12 | -0.04 | -0.20 | -0.02 | -0.06 | -0.02 | -0.17* |
| TpTe | *0.00* | -0.16 | 0.06 | -0.20* | -0.39** | 0.01 | -0.07 | -0.27** | -0.10 |
| *f* | *0.00* | 0.11 | 0.22* | 0.31*** | 0.31* | 0.14 | 0.16 | 0.25** | -0.07 |
| T_I_ | *-0.17* | -0.16 | 0.20 | -0.38*** | -0.29* | -0.16 | -0.26** | -0.26** | -0.05 |
| T_E_ | *-0.01* | -0.09 | -0.21* | -0.33*** | -0.17 | -0.04 | -0.20* | -0.18* | 0.08 |
| T_T_ | -0.08 | -0.10 | -0.17 | -0.41*** | -0.37** | -0.19 | -0.24** | -0.23** | 0.02 |
| Pause | 0.55*** | -0.33*** | -0.12 | **-**0.02 | -0.32* | 0.02 | -0.02 | -0.31*** | -0.05 |
| PM_2.5_ mass | -0.01 | 0.12 | 0.07 | -0.01 | -0.05 | -0.13 | 0.04 | -0.06 | -0.12 |
| PM_2.5_ count | 0.04 | 0.12 | -0.02 | 0.01 | -0.05 | -0.12 | -0.05 | 0.05 | -0.14 |
| NO | 0.00 | 0.13 | 0.06 | -0.01 | -0.03 | -0.09 | 0.02 | -0.01 | -0.04 |
| NO_x_ | -0.08 | 0.10 | 0.10 | -0.07 | -0.06 | -0.14 | 0.10 | -0.01 | -0.15 |
| O_3_ | -0.01 | 0.04 | -0.08 | 0.01 | -0.04 | -0.07 | -0.06 | 0.03 | -0.13 |
| CO | -0.08 | 0.02 | 0.01 | -0.07 | -0.05 | -0.10 | 0.02 | -0.01 | -0.17 |

**Supplemental Table 5.** Pearson correlation coefficients derived from all MetS rats’ daily changes in physiologic or pollutant variables and log-transformed changes in arrhythmia count from baseline. Pearson partial correlation was conducted for pollutant concentrations. *, **, and *** indicate P < 0.05, 0.01, and 0.001, respectively. AVB: AV Block, Adv.: advanced, SAB: sinoatrial block, VPB: ventricular premature beat, A-tach: atrial tachycardia (≥ 3 consecutive premature beats), V-tach: ventricular tachycardia, pNN15: percentage of normal RR intervals > 15 ms different from prior RR interval, SBP: systolic BP, DBP: diastolic BP, MBP: mean BP, PP: pulse pressure, QTcB: Bazzett's HR- corrected QT, QTcF: Fridericia's HR-corrected QT, QTe: Q to T-end interval, RPPP: rate-pressure product.

|  | *f* | T_T_ | T_E_ | T_I_ | Pause |
| --- | --- | --- | --- | --- | --- |
| RMSSD | -0.27** | 0.21* | 0.17 | 0.03 | 0.18 |
| HF | -0.23* | 0.20* | 0.16 | 0.06 | 0.08 |
| SDNN | 0.04 | -0.13 | 0.00 | -0.23** | 0.24** |

**Supplemental Table 6. Pearson Correlation of HRV with Respiratory Physiology.** *, **, and *** indicate P < 0.05, 0.01, and 0.001, respectively.

**Supplemental Figure 3.** Mean AV block Mobitz type I events (± SE) in MetS rats during baseline and exposure to P+SOA (solid squares) or FA (open circles). Vertical lines delineate exposure weeks. BL: baseline.

References

1. Chow JC, Watson JG, Pritchett LC, Pierson WR, Frazier CA, Purcell RG: **The Dri Thermal Optical Reflectance Carbon Analysis System - Description, Evaluation and Applications in United-States Air-Quality Studies**. *Atmos Environ a-Gen* 1993, **27**(8):1185-1201.

2. Souza N, Vanderlei L, Garner DM: **Risk evaluation of diabetes mellitus by relation of chaotic globals to HRV**. *Complexity* 2015, **20**:84-92.

3. Wajnsztejn F, Vanderlei L, de Abreu L, Garner D: **Heart rate variability analysis by chaotic global techniques in children with attention deficit hyperactivity disorder**. *Complexity* 2015, **21**:412-419.

4. Razali N, Wah YB: **Power comparisons of shapiro-wilk, kolmogorov-smirnov, lilliefors and anderson-darling tests**. *J Statist Modeling and Analytics* 2011, **2**:21-33.

5. Khoa TQ, Ha VQ, Toi VV: **Higuchi fractal properties of onset epilepsy electroencephalogram**. *Comput Math Methods Med* 2012, **2012**:Article # 461426.

6. Vanderlei FM, Vanderlei LCM, Carlos de Abreu L, Garner DM: **Entropic Analysis of HRV in Obese Children**. *Int Arch Med* 2015, **8**(200):1-9.
